# Trainee performance in radical gastrectomy and its effect on outcomes

**DOI:** 10.1002/bjs5.50219

**Published:** 2019-11-06

**Authors:** M. Navidi, A. Madhavan, S. M. Griffin, P. Prasad, A. Immanuel, N. Hayes, A. W. Phillips

**Affiliations:** ^1^ Northern Oesophago‐Gastric Unit, Royal Victoria Infirmary Queen Victoria Road, Newcastle upon Tyne NE1 4LP UK

## Abstract

**Background:**

This study aimed to determine whether trainee involvement in D2 gastrectomies was associated with adverse outcomes.

**Methods:**

Data from a prospectively created database of consecutive patients undergoing open D2 total (TG) or subtotal (STG) gastrectomy with curative intent between January 2009 and January 2014 were reviewed. Short‐ and long‐term clinical outcomes were compared in patients operated on by consultants and those treated by trainees under consultant supervision.

**Results:**

A total of 272 D2 open gastrectomies were performed, 123 (45·2 per cent) by trainees. There was no significant difference between consultants and trainees in median duration of surgery (TG: 240 (range 102–505) *versus* 240 (170–375) min respectively, *P* = 0·452; STG: 225 (150–580) *versus* 212 (125–380) min, *P* = 0·192), number of resected nodes (TG: 30 (13–101) *versus* 30 (11–102), *P* = 0·681; STG: 26 (5–103) *versus* 25 (1–63), *P* = 0·171), length of hospital stay (TG: 15 (7–78) *versus* 15 (8–65) days, *P* = 0·981; STG: 10 (6–197) *versus* 14 (7–85) days, *P* = 0·242), overall morbidity (TG: 44 *versus* 49 per cent, *P* = 0·314; STG: 34 *versus* 25 per cent, *P* = 0·113) or mortality (TG: 4 *versus* 2 per cent; *P* = 0·293). No difference in predicted 5‐year overall survival was noted between the two cohorts (TG: 68 per cent for consultants *versus* 77 per cent for trainees, *P* = 0·254; STG: 70 *versus* 75 per cent respectively, *P* = 0·512). The trainee cohort had lower median blood loss for both TG (360 (range 90–1200) ml *versus* 600 (70–2350) ml for consultants; *P* = 0·042) and STG (235 (50–1000) *versus* 360 (50–3000) ml respectively; *P* = 0·053).

**Conclusion:**

Clinical outcomes were not compromised by supervised trainee involvement in D2 open gastrectomy.

## Graphical abstract

This study of 272 D2 gastrectomies investigated whether trainee involvement was associated with adverse outcomes. No significant difference in morbidity, mortality, operating time, number of nodes resected, hospital stay or predicted 5‐year survival was noted, demonstrating that outcomes are not compromised by supervised trainee involvement.

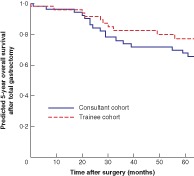

Equally good performance

## Abstract (es)

**Antecedentes:**

El hecho de que en operaciones complejas la experiencia quirúrgica sea limitada puede influir en los resultados. Esto puede ser especialmente relevante cuando estas operaciones son realizadas por cirujanos en formación bajo supervisión. El objetivo de este estudio fue determinar si la participación del cirujano en formación en las gastrectomías D2 se asociaba con resultados adversos.

**Métodos:**

Se revisó la información recogida en una base de datos prospectiva de pacientes consecutivos sometidos a gastrectomía D2 abierta total (*total gastrectomy*, TG) o subtotal (*subtotal gastrectomy*, STG) con intención curativa desde enero de 2009 a enero de 2014. Los pacientes se dividieron en dos grupos, uno de pacientes operados por un cirujano consultor y otro, de pacientes operados por un cirujano en periodo formación bajo la supervisión de un cirujano consultor. Se compararon los resultados clínicos a corto y largo plazo incluyendo la supervivencia global esperada a los cinco años.

**Resultados:**

Se realizaron un total de 272 gastrectomías D2 abiertas (45% por cirujanos en periodo de formación). Las características demográficas de los pacientes fueron similares en los grupos de los cirujanos en formación y cirujanos consultores. En la TG y STG, no se apreciaron diferencias significativas entre ambas cohortes en el tiempo operatorio (*P* = 0,45)y (*P* = 0,19), número de ganglios linfáticos extirpados (*P* = 0,68) y (*P* = 0,17), duración de la estancia hospitalaria (*P* = 0,98) y (*P* = 0,24), morbilidad global (*P* = 0,31) y (*P* = 0,11), mortalidad (*P* = 0,29) y supervivencia global esperada a los 5 años (*P* = 0,25) y (*P* = 0,51). La pérdida sanguínea en ambas TG y STG fue menor en la cohorte de cirujanos en formación (*P* < 0,05).

**Conclusión:**

La práctica de una gastrectomía D2 abierta por cirujanos en periodo de formación supervisados por consultores no comprometían los resultados clínicos.

## Introduction

Trainee involvement in surgical procedures has been associated with benefit as well as adverse outcomes^1^, ^2^, ^3^, ^4^. Studies have suggested that trainee involvement can result in improved colonic cancer detection rates at endoscopy^5^, lower bleeding complication rates in endocrine surgery^6^, and lower morbidity levels at teaching hospitals compared with non‐teaching hospitals^7^. Other studies^1^, ^8^, however, have suggested that trainee involvement may be detrimental. A recent study^9^ has suggested that the grade of trainee may be an important factor, with senior trainee involvement associated with poorer outcomes. Two studies^10^, ^11^ of trainee involvement in oesophagectomy, where trainees were likely to be senior, demonstrated no adverse impact on outcomes.

Changes in working patterns for trainees in many countries have meant increasing pressures on time available for training, and a shift towards achieving ‘competency’^12^, ^13^. Although the aim of these changes was to improve working conditions for surgical trainees, the potential adverse impact of reduction in hours is to reduce operative experience. Therefore, the challenge to provide excellent and evidence‐based patient‐centred care, while maintaining a viable surgical training programme, has become an important challenge in the face of decreasing training hours^14^, ^15^.

Radical gastrectomy with systematic lymphadenectomy (often referred to as D2 gastrectomy) for adenocarcinoma of the stomach is a technically demanding operation associated with significant morbidity^16^. A recent study^17^ demonstrated that the current level of active exposure of surgical trainees to laparoscopic gastric surgery for gastric cancer may be insufficient. Two other studies^18^, ^19^ indicated that less than 30 per cent of laparoscopic gastrectomies are carried out by trainees. Both studies demonstrated similar blood loss, lymph node harvest and short‐term morbidity between trainees and trainers as primary surgeons. In one of these studies^18^, trainees took longer to perform the procedure.

As there appear to be no published data on the impact of trainee involvement on outcomes, including long‐term survival, following open radical gastrectomy for adenocarcinoma, this study was undertaken to determine whether trainee involvement as the primary surgeon performing open D2 gastrectomies affected short‐ and long‐term surgical outcomes.

## Methods

A prospectively developed database of all patients undergoing a radical D2 open gastrectomy with curative intent for malignant disease was reviewed. Data from patients treated at a single centre (Northern Oesophago‐Gastric Unit, Newcastle upon Tyne, UK) between January 2009 and January 2014 were analysed. All patients were discussed by the multidisciplinary team following adequate staging investigations, and subsequently had an open total (TG) or subtotal (STG) gastrectomy with D2 lymphadenectomy, either as unimodality treatment or following neoadjuvant chemotherapy^20^.

Data including baseline demographics (age, sex, stage of disease, use of neoadjuvant treatment) were collected. Immediately after surgery, ASA grade, blood loss, duration of surgery, and grade of the primary surgeon and surgical assistants were recorded. Data collection was completed upon discharge, with details of final histology, stage of disease, lymph node yield, length of hospital stay and complications recorded. Data were entered by the consultant and trainee, and checked by an independent data manager. Complications were recorded and graded according to the Expanded Accordion Severity Classification of Postoperative Complications^21^. The UICC TNM (7th edition) staging system^22^ was used for all patients.

Resections were carried out using a standard open approach with a radical *en bloc* D2 lymphadenectomy. Proximal tumours and patients diagnosed with diffuse‐type gastric cancers were treated by TG. STG was used for patients with a distal tumour where adequate clearance could be achieved. For both operations, resection and reconstruction phases were performed in an identical manner by consultants and trainees. TG involved an oesophagojejunal retrocolic stapled anastomosis fashioned with a circular stapling device, creating an end‐to‐side anastomosis. The small bowel end was closed with a stapler. Continuity with a 45‐cm Roux limb was created by a two‐layered continuous end‐to‐side handsewn anastomosis. For STG, the stomach was transected with a stapling device, the Roux limb was prepared as above, and an end‐to‐side gastrojejunostomy was created with a handsewn, two‐layered anastomosis.

### Analysis

Patients were divided into two cohorts according to whether a consultant or a trainee primarily performed the procedure. The trainee was regarded as being the primary surgeon if they performed more than 50 per cent of the operation. This would, in practice, mean that the trainee either performed all the steps described in the surgical technique section, or at least that the critical steps were performed by the trainee, such as the resection and lymphadenectomy. This was documented immediately after the operation, and agreed between trainer and trainee.

All documented complications were rechecked at monthly morbidity and mortality meetings to ensure a consensus on complication classification, and a final check was carried out by an independent data manager.

### Statistical analysis

Kruskal–Wallis and Mann–Whitney *U* tests were used to compare continuous variables, and categorical data were compared with χ^2^ or Fisher's exact test. Median (range) values are presented for descriptive data. The log rank (Mantel–Cox) test was used to compare survival between the groups. *P* < 0·050 (two‐sided) was considered statistically significant. Statistical analysis was performed using SPSS® version 22.0 (IBM, Armonk, New York, USA).

## Results

Between January 2009 and January 2014, 167 patients underwent D2 STG and 105 had a TG. There were six consultants providing care and nine trainees, all of whom were within the last 3 years of training and had undertaken a median of 9 (range 0–35) malignant upper gastrointestinal resections before commencement of training at this institution.

### Subtotal gastrectomy

A total of 95 resections were performed by the six consultants and 72 by the nine trainees. No significant difference was observed in patient demographics, tumour histology, stage or ASA grade between the cohorts (*Table* 1). There was no difference between consultant and trainee cohorts in median duration of surgery (225 (range 150–580) *versus* 212 (125–380) min respectively; *P* = 0·192)*,* number of resected nodes (26 (5–103) *versus* 25 (1–63); *P* = 0·171) or length of hospital stay (10 (6–197) *versus* 14 (7–85) days; *P* = 0·242). However, there was lower median blood loss in the trainee cohort (235 (50–1000) ml *versus* 360 (50–3000) ml for consultants; *P* = 0·053). Major morbidity was significantly higher in the consultant cohort (12 per cent *versus* 4 per cent in the trainee cohort; *P* = 0·043). There was no significant difference in overall morbidity, or in the predicted 5‐year overall survival rate (70 per cent in the consultant cohort *versus* 75 per cent in the trainee cohort; *P* = 0·514).

**Table 1:** Characteristics and outcomes of patients in the subtotal gastrectomy group

	Consultant cohort (*n* = 95)	Trainee cohort (*n* = 72)	*P* †
**Age (years)** *	72 (24–90)	74 (28–86)	0·166‡
**Sex ratio (M** : **F)**	57 : 38	40 : 32	0·189
**Histology**			0·821
Adenocarcinoma	93	70	
Other	2	2	
**Neoadjuvant therapy**			0·032
None	64 (67)	58 (81)	
Chemotherapy	31 (33)	14 (19)	
**Adjuvant therapy**			0·771
None	84 (88)	63 (88)	
Chemotherapy	11 (12)	9 (12)	
**BMI (kg/m** ^**2**^ **)** *	26 (18–44)	24·5 (17–39)	0·914‡
**ASA grade**			0·354
I	6	4	
II	45	36	
III	41	31	
IV	3	1	
**pT category**			0·832
pT1	23	13	
pT2	14	12	
pT3	32	26	
pT4	24	18	
pTx (HGD)	2	3	
**pN category**			0·954
pN0	41	30	
pN1	23	17	
pN2	17	12	
pN3	14	13	
**Duration of surgery (min)** *	225 (150–580)	212 (125–380)	0·192‡
**No. of resected nodes** *	26 (5–103)	25 (1–63)	0·171‡
**Blood loss (ml)** *	360 (50–3000)	235 (50–1000)	0·053‡
**Complications (all)**	32 (34)	18 (25)	0·113
**Major complications (Accordion level ≥ 3)**	11 (12)	3 (4)	0·043
**In‐hospital mortality**	0	0	
**Length of hospital stay (days)** *	10 (6–197)	14 (7–85)	0·242‡

Values in parentheses are percentages unless indicated otherwise;

* values are median (range). HGD, high‐grade dysplasia.

† χ^2^ or Fisher's exact test, except

‡ Kruskal–Wallis or Mann–Whitney *U* test.

### Total gastrectomy

A total of 54 resections were performed by the six consultants and 51 by the nine trainees.

No significant difference was noted in the demographics between the two cohorts (*Table* 2). Again, there was no significant difference between consultant and trainee cohorts in the operative outcome data with regard to median duration of surgery (240 (range 102–505) *versus* 240 (170–375) min respectively; *P* = 0·452), number of resected nodes (30 (13–101) *versus* 30 (11–102); *P* = 0·681) or length of hospital stay (15 (7–78) *versus* 15 (8–65) days; *P* = 0·981). The trainee cohort had a significantly lower median blood loss (360 (90–1200) ml *versus* 600 (70–2350) ml in the consultant cohort; *P* = 0·042). There was no significant difference in overall and major morbidity between the two cohorts, or in the predicted 5‐year overall survival rate (68 per cent in the consultant cohort *versus* 77 per cent in the trainee cohort; *P* = 0·254).

**Table 2:** Characteristics and outcomes of patients in the total gastrectomy group

	Consultant cohort (*n* = 54)	Trainee cohort (*n* = 51)	*P* †
**Age (years)** *	67 (41–81)	72 (43–87)	0·062‡
**Sex ratio (M** : **F)**	41 : 13	31 : 20	0·464
**Histology**			0·054
Adenocarcinoma	46	50	
Other	8	1	
**Neoadjuvant therapy**			0·484
None	26 (48)	29 (57)	
Chemotherapy	28 (52)	22 (43)	
**Adjuvant therapy**			0·882
None	46 (85)	43 (84)	
Chemotherapy	8 (15)	8 (16)	
**BMI (kg/m** ^**2**^ **)** *	27 (18–43)	25 (20–46)	0·384‡
**ASA grade**			0·474
I	2	2	
II	38	32	
III	14	16	
IV	0	1	0·343
**pT category**			
pT1	10	8	
pT2	12	15	
pT3	16	20	
pT4	11	7	
pTx (HGD)	5	1	
**pN category**			0·791
pN0	26	23	
pN1	10	13	
pN2	10	7	
pN3	8	8	
**Duration of surgery (min)** *	240 (102–505)	240 (170–375)	0·452‡
**No. of resected nodes** *	30 (13–101)	30 (11–102)	0·681‡
**Blood loss (ml)** *	600 (70–2350)	360 (90–1200)	0·042‡
**Complications (all)**	24 (44)	25 (49)	0·313
**Major complications (Accordion level ≥ 3)**	8 (15)	5 (10)	0·222
**In‐hospital mortality**	2 (4)	1 (2)	0·293
**Length of hospital stay (days)** *	15 (7–78)	15 (8–65)	0·981‡

Values in parentheses are percentages unless indicated otherwise;

* values are median (range). HGD, high‐grade dysplasia.

† χ^2^ or Fisher's exact test, except

‡ Kruskal–Wallis or Mann–Whitney *U* test.

## Discussion

This study showed that patient outcomes were not affected adversely by a supervised trainee performing a radical total or subtotal gastrectomy. Short‐ and long‐term outcomes were comparable between operations performed by consultants and trainees. The consultant cohort had greater blood loss for both procedures, and a higher rate of major complications in the STG cohort, although neither of these features affected length of hospital stay or predicted survival. A recent study^6^ of more than 80 000 patients undergoing thyroid and parathyroid surgery demonstrated a decrease in the odds of bleeding complications in the trainee cohort. It is difficult to determine accurately the reasons for the discrepancy in blood loss in both operations, and also morbidity in the STG group. It may be that procedures in some patients, anticipated as being more difficult, were either consciously or subconsciously selected by trainers as less suitable for a trainee to perform. It is also possible that when a problem was encountered in a procedure initially undertaken by a trainee, the operation was taken over by a consultant. It was not possible to determine the number of occasions when this occurred, as no record was kept of any decision on who commenced a procedure and was intended to be the primary surgeon.

Previous studies have demonstrated higher rates of complications in emergency general surgical procedures undertaken by trainees^2^, as well as longer time taken by trainees to perform gastrectomies^18^ and gastric bypass procedures^23^. The present study, however, demonstrated no inferiority in the rate of overall complications for both STG and TG, or in the duration of surgery between trainees and consultants. This finding is supported by previous work^11^, ^24^ in foregut surgery, including oesophageal, pancreatic and hepatic surgery, in which no compromise to patient outcomes was noted when a trainee was the primary surgeon. Other surgical specialties, including colorectal surgery, non‐oesophageal thoracic (lobectomies) and coronary artery bypass surgery, have also demonstrated non‐inferiority of outcomes when trainees or residents were the primary surgeons^25^, ^26^, ^27^.

A limitation of the study is its retrospective nature. It is not possible to determine how consultants decided which operations were suitable for trainees. In the absence of difference between the cohorts in ASA grade, BMI or stage of disease, it may be that trainers chose to perform the procedures they anticipated as being the most challenging. This may account for the greater blood loss in the trainer cohorts for both TG and STG.

Informed patient consent forms a fundamental part of the preoperative dialogue between surgeons and patients. Patients must be fully informed that a trainee may perform part or all of a procedure supervised. A previous study^28^ suggested that as patients are made aware more explicitly of the involvement of trainees in a procedure, they are less likely to consent. The present study allows surgeons to provide evidence to patients that trainee involvement in a radical gastrectomy does not compromise short‐ or long‐term outcomes.

## Disclosure

The authors declare no conflict of interest.
